# A Rare and Severe Presentation of Henoch-Schönlein Purpura in an Adolescent With Crescentic Glomerulonephritis, Arrhythmia, Acute Gastrointestinal Bleed, and Neurological Complications

**DOI:** 10.7759/cureus.14169

**Published:** 2021-03-29

**Authors:** Siddharth Shah, Jessica Hata

**Affiliations:** 1 Pediatric Nephrology, Norton Children's Hospital, Louisville, USA; 2 Pathology, Norton Children's Hospital, Louisville, USA

**Keywords:** hsp, gastrointestinal bleed, glomerulonephritis, cardiac arrythmia

## Abstract

Henoch-Schönlein purpura (HSP) is a childhood vasculitis disorder that involves the skin, joints, gastrointestinal (GI) tract, and kidneys. It is related to immunoglobulin A (IgA) antibody deposition in small blood vessels. HSP is a self-limiting disorder, but its morbidity is primarily associated with renal involvement. GI pathologies like intussusception, gastritis, duodenitis, ileitis, or ulcer have been reported to be associated with this disease. However, cardiac and neurological complications are rarely reported.

We present the case of a 16-year-old, previously healthy male who was diagnosed with HSP after presenting with a non-blanching purpuric rash in the lower extremities. The patient also had joint and abdominal pain, and swelling in the extremities. There was renal dysfunction at presentation with blood urea nitrogen (BUN) of 67 mg/dL and serum creatinine of 1.9 mg/dL. The serum albumin was low at 2 g/dL, and the patient had nephrotic range proteinuria. Urine microscopy showed red blood cell casts. A renal biopsy was performed, which showed IgA deposition in glomeruli. He was started on intravenous (IV) pulse methylprednisolone and was later prescribed oral steroids. Four weeks after the treatment initiation, he presented with syncope and acute anemia (hemoglobin of 3.5 g/dL). The fecal occult blood was positive. Esophagogastroduodenoscopy (EGD) was not suggestive of gastritis, duodenitis, or ulcer. The pill-cam capsule endoscopy revealed GI bleeding from the terminal ileum near Meckel’s diverticulum. He subsequently required blood transfusions, and the bleeding eventually improved with symptomatic management. Six weeks after treatment initiation, he presented with dizziness and palpitations. The EKG showed the presence of atrial fibrillation, and he had an episode of non-sustained ventricular tachycardia on telemetry. Arrhythmia was diagnosed secondary to HSP cardiac vasculitis, and we initiated treatment with metoprolol and amiodarone. Seven weeks after the initial treatment, he had neurological clinical findings of proximal muscle weakness, tremors, and upper and lower extremity clonus. A second renal biopsy was then performed due to the presence of persistently elevated serum creatinine, which showed 75% of glomeruli with cellular crescents. He was treated with IV cyclophosphamide. Subsequently, the renal function improved. There were no other GI, cardiac, or neurological complications after six months of follow-up.

The presentation of HSP can be more severe in adolescents, and they need to be closely monitored for GI, cardiac, renal, and neurological complications after the disease occurrence. Bleeding from Meckel’s diverticulum or an episode of non-sustained ventricular tachycardia with HSP has not been previously reported to our knowledge. Arrhythmia is an exceptionally unusual occurrence in HSP, and it is usually treated with anti-arrhythmic drugs and intensification of the immunosuppressive regimen.

## Introduction

Henoch-Schönlein purpura (HSP) is a leukocytoclastic vasculitis involving small blood vessels. It is typically seen in children and characterized by the deposition of immunoglobulin A (IgA) antibodies in small blood vessels. The condition usually involves organs such as the skin, joints, gastrointestinal (GI) tract, and kidneys [1-3]. The annual incidence of HSP varies and ranges from 10 to 30 cases per 100,000 children. The median age of onset is four to five years and is predominantly seen in children less than 10 years of age. It has a male preponderance (male-to-female ratio: 1.4-1.7:1), frequently presents in colder months, and is usually triggered after respiratory viral infections [1-3].

HSP usually presents with clinical features of skin rash, GI symptoms, arthritis or arthralgia, and kidney diseases of varying severity [1-3]. The skin rash is a typical finding in all patients with HSP. It consists of petechia or palpable purpura that does not disappear with pressure and involves the extensor surface of lower limbs, buttocks, and forearms with occasional involvement of trunk and face [1]. Arthritis or arthralgia is present in nearly three-fourth of the affected children, and it is typically oligo-articular, involving large joints of lower extremities [1]. GI symptoms are found in almost two-thirds of patients presenting with HSP and include diffuse abdominal pain associated with meals, as well as nausea and vomiting [1]. The GI complications include visible GI bleed (hematemesis and melena), intussusception, ulcer, rare instances of intestinal infarction, bowel perforation, and gangrene [1,3]. The kidneys are involved in 20-55% of children with HSP, and renal involvement ranges from isolated hematuria, hematuria, and proteinuria to acute nephritic or nephrotic syndrome [1,3]. In rare cases, children can present with rapidly progressive glomerulonephritis [2-3]. While the disease is self-limiting, renal involvement is the primary cause of disease morbidity [2]. Other features reportedly associated with HSP include neurological complications from cerebral vasculitis such as convulsion, encephalopathy or chorea, pulmonary-renal syndrome, adrenal bleeding, testicular involvement presenting as torsion, or ureteral stenosis [3]. HSP complicated by cardiac involvement is rarely reported in the literature but can be life-threatening [4]. This report aims to discuss severe renal, cardiac, GI, and neurological complications that can occur with HSP recurrence in pediatric patients, particularly adolescents, and highlights the need to monitor the patients closely after the initial diagnosis.

## Case presentation

Patient information

Our patient was a 16-year-old male with no significant past medical history who presented initially with a skin rash on the lower extremities, lower extremity joint pain, and left knee swelling. He was prescribed oral steroids at his pediatrician’s office, but the symptoms did not improve. Also, he started having abdominal pain a few days later, which was diffuse, constant, and limited his oral intake. He also had nausea, vomiting, blood-streaked stools, and dark brown urine. Two weeks after the onset of symptoms, he presented to our tertiary hospital, and he was admitted. There was no history of the use of non-steroid anti-inflammatory drugs (NSAIDs) before the presentation.

Clinical findings

**Figure 1:**
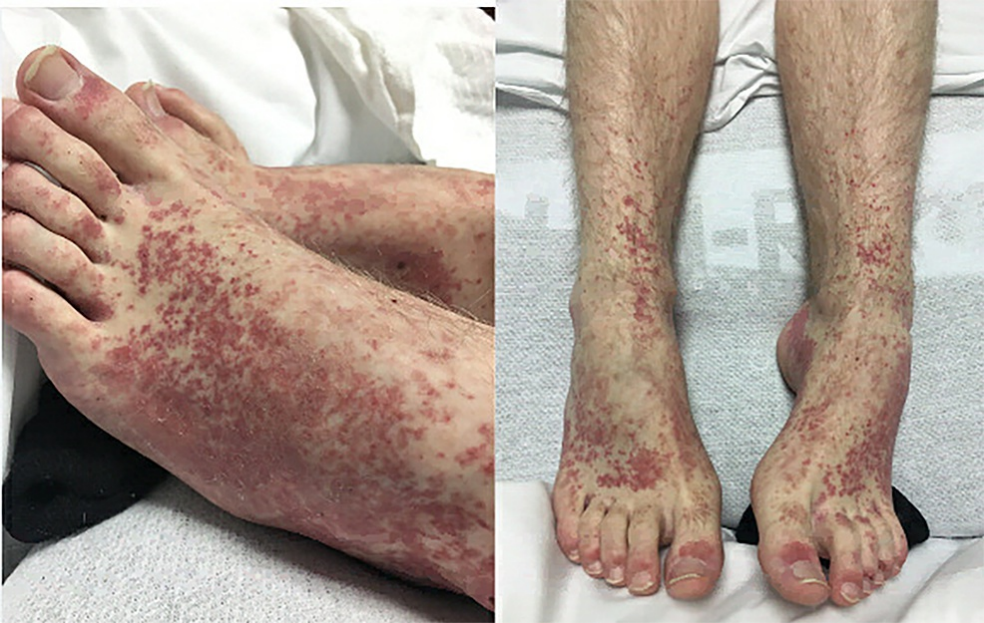
Skin rash on extremities

He was afebrile at admission with a BP of 136/60 mmHg, and his bodyweight at admission was 71 kg. He had lost 7 kg of weight in two weeks since the onset of illness. He had diffuse, erythematous, non-blanching, purpuric skin rash involving the extensor surface of lower extremities on physical exam (Figure 1).

He also had tenderness and mild swelling on the left knee. The initial lab results were significant for a white blood cell (WBC) count of 19 × 10^3^/μl and hemoglobin of 10.7 g/dL; the platelet count was normal at 336 × 10³/μl. There was significant renal dysfunction at presentation with a blood urea nitrogen (BUN) level of 74 mg/dL (reference range: 7-20 mg/dL) and serum creatinine of 2.4 mg/dL (reference range: 0.3-1.2 mg/dL). The serum albumin was low at 2.4 g/dL (reference range: 3.5-5 g/dL). The C-reactive protein (CRP) was 17.1 mg/dL (reference range: <1 g/dL). The urinalysis showed large amounts of blood, 300 mg/dL of protein, several red blood cell casts/high power field on urine microscopy, and the urine protein and creatinine ratios were high at 6.4 (reference range: <0.2). The stool occult blood-guaiac was positive. The abdominal ultrasound (US) was negative for intussusception. The renal US showed bilateral echogenic kidneys with kidney sizes of 11.9 cm on the right kidney and 12.3 cm on the left kidney.

Diagnostic assessment

Infectious studies, including stool culture and blood culture, were obtained at presentation and were negative. The tick-borne disease antibody panel was also negative. Glomerulonephritis was diagnosed based on the urinary findings, elevated BP, and high BUN and serum creatinine. The differentials of glomerulonephritis, such as post-infectious glomerulonephritis, lupus, and vasculitis, were considered. The immunological workup showed normal ranges of complement C3 and C4. The anti-nuclear antibody (ANA) screen was negative. The anti-nuclear cytoplasmic antibody (ANCA) screen revealed negative myeloperoxidase immunoglobulin G (IgG) and negative proteinase-3 IgG.

We diagnosed the presence of HSP based on the clinical presentation of skin rash, diffuse abdominal pain, joint symptoms, and significant renal involvement. Given the high BUN and serum creatinine, we were also concerned about the presence of rapidly proliferative and crescentic glomerulonephritis. The patient underwent a renal biopsy one day after the admission, which showed IgA nephropathy with no cellular crescents, but the glomeruli were hypercellular.

Therapeutic assessment

We started intravenous (IV) pulse steroid therapy (methylprednisolone) at admission to treat HSP-associated inflammation and kidney disease, beta-blockers (labetalol) to treat blood pressure, and IV proton-pump inhibitors for gastroprotection. The rationale for starting IV steroid therapy was related to a severe degree of glomerulonephritis seen at presentation. The serum creatinine levels improved to 1.5 mg/dL after three days, and we initiated oral steroid (prednisone) treatment at a dose of 30 mg twice a day. There was an improvement in joint symptoms; however, the patient continued to have abdominal pain and diarrhea for one week after treatment initiation. He was discharged home with instructions for close follow-up in a pediatric nephrology clinic. The hemoglobin level was 10.1 g/dL at the time of discharge.

Follow-up and outcomes

**Figure 2:**
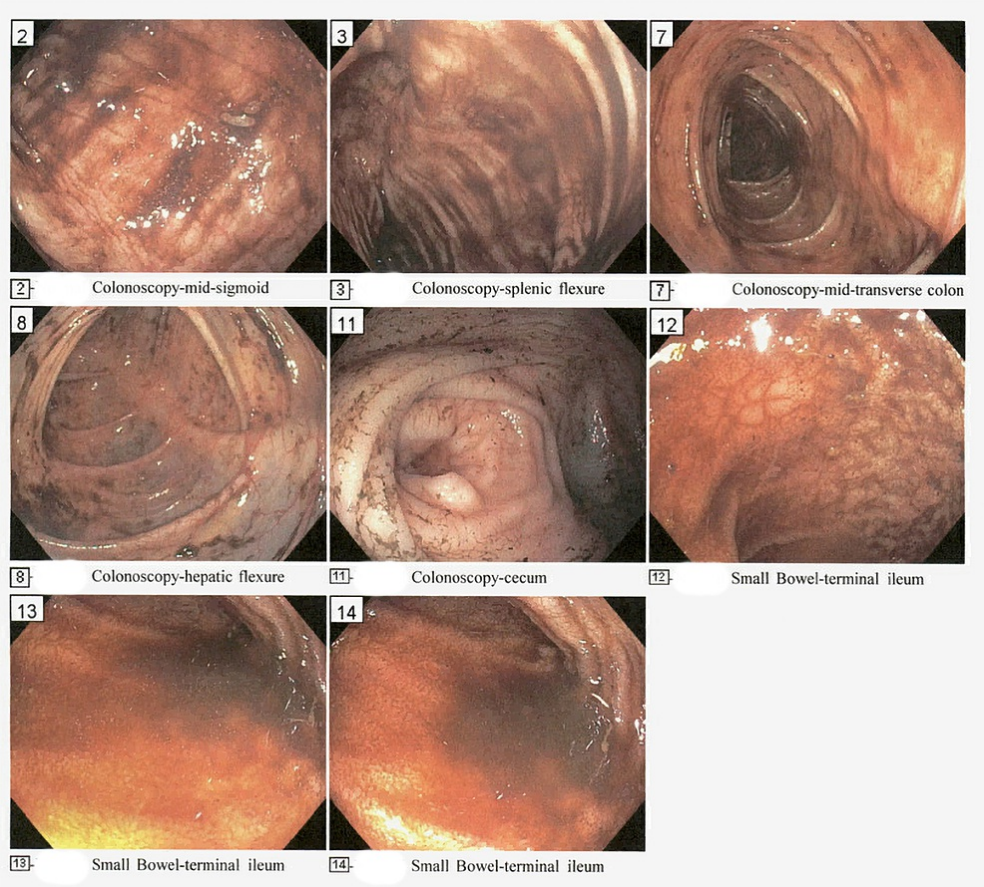
Colonoscopy images Colonoscopy showed lower GI tract bleeding GI: gastrointestinal

Four weeks after the treatment initiation with steroids, the patient presented to the ER with bright red stool and syncope. The stool guaiac was positive. The hemoglobin was very low at 3.5 g/dL, and he was suspected of having symptomatic anemia secondary to GI bleed. The BUN level was 78 mg/dL, and serum creatinine was 1.4 mg/dL at presentation. The elevation in BUN may also have been contributed by GI bleed. The patient required blood transfusions. Esophagogastroduodenoscopy (EGD) and colonoscopy with biopsy were performed. Surprisingly, the EGD did not show active bleed. However, the colonoscopy showed dark fluid in the colon's lumen, raising the possibility of a more proximal or ileal source of bleeding (Figure 2).

The biopsy showed mild ileitis. Pill-cam capsule endoscopy was subsequently performed, which showed the bleeding from the terminal ileum, and the presence of Meckel's diverticulum adjacent to it was detected. However, the GI bleeding had significantly improved by the time the capsule endoscopy was done, and the hemoglobin remained stable without further blood transfusions. Surgery service was consulted, but given the risk of surgical complications with HSP vasculitis involving the GI tract and the rapid improvement in GI bleeding, surgery was not performed. The patient was closely monitored, and there was no major recurrence of lower GI bleeding.

**Figure 3:**
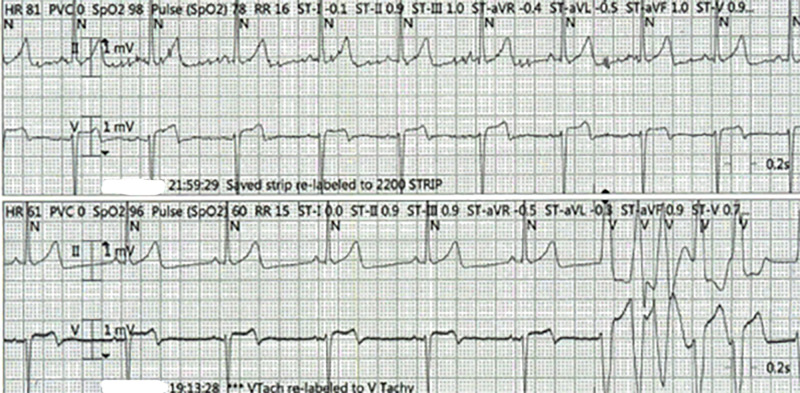
Telemetry recording showing non-sustained ventricular tachycardia

Six weeks after the initial treatment with steroids, the patient presented with dizziness, lightheadedness, and palpitations. In the ER, the EKG was suggestive of atrial fibrillation with accelerated ventricular rhythm. While he was on telemetry, the patient had another arrhythmia episode, now showing non-sustained ventricular tachycardia (Figure 3).

The troponins were elevated at 0.101 ng/mL (reference range: 0-0.034 ng/mL). The echocardiogram showed good biventricular function. Even though previous reports of arrhythmia secondary to HSP were rare, a diagnosis of HSP-induced arrhythmia was made based on elevated troponins and arrhythmia patterns. He was started on amiodarone and metoprolol, and there were no further recurrences of arrhythmia.

**Figure 4:**
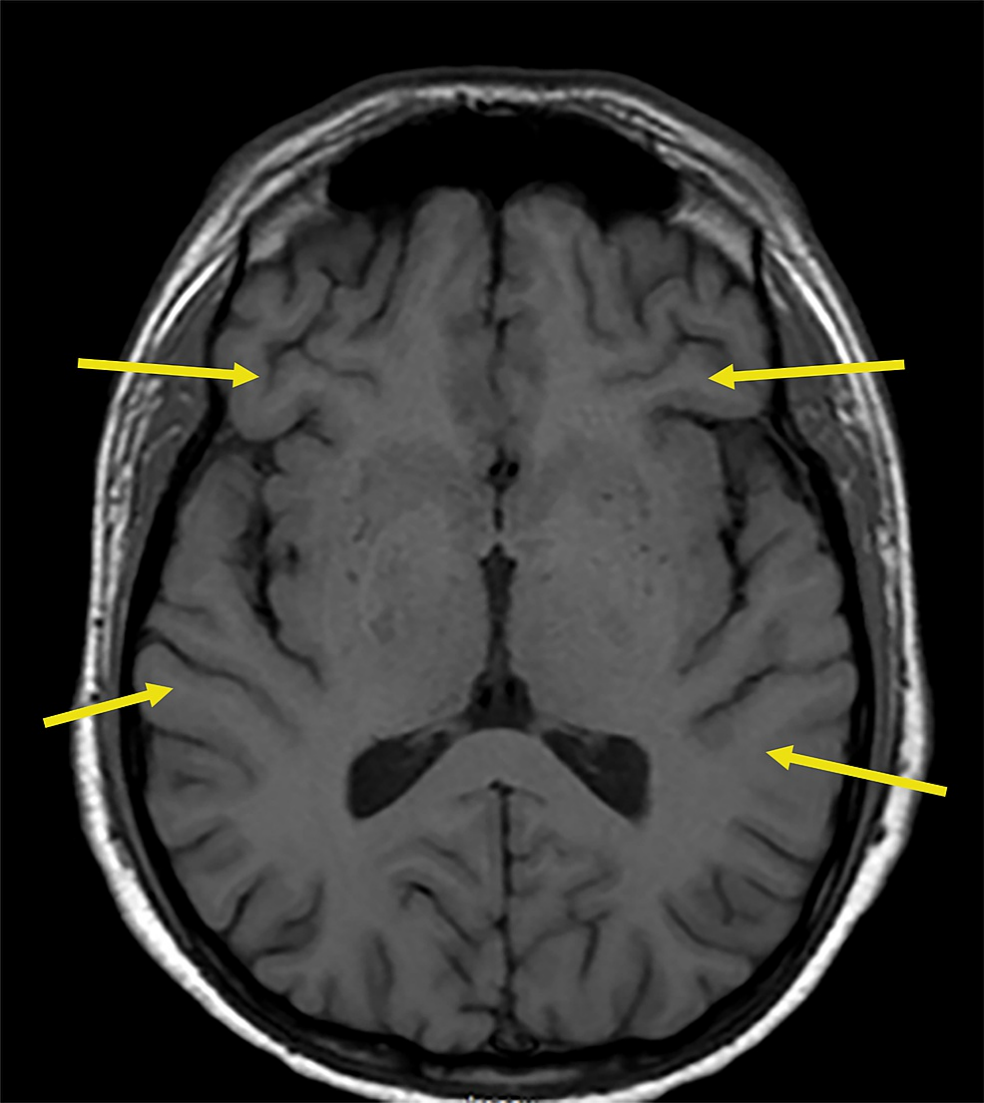
Brain MRI showing sulcal prominence (arrows) MRI: magnetic resonance imaging

Seven weeks after initial treatment with steroids, there was an elevation in serum creatinine, and new-onset neurological findings were observed. These neurological findings included the presence of tremor, gait disturbance, proximal muscle weakness, bilateral ankle and knee clonus, upper extremity clonus with positive Hoffman sign, and generalized hyperreflexia. The tremor was present in the tongue as well as both extremities. These findings were suspected to be secondary to the central nervous system (CNS) vasculitis, steroids, or amiodarone. The MRI of the brain showed diffuse sulcal prominence (Figure 4).

The cerebrospinal fluid (CSF) exam was negative for infection. The autoimmune encephalitis antibody panel was also negative. The patient was treated with clonazepam.

**Figure 5:**
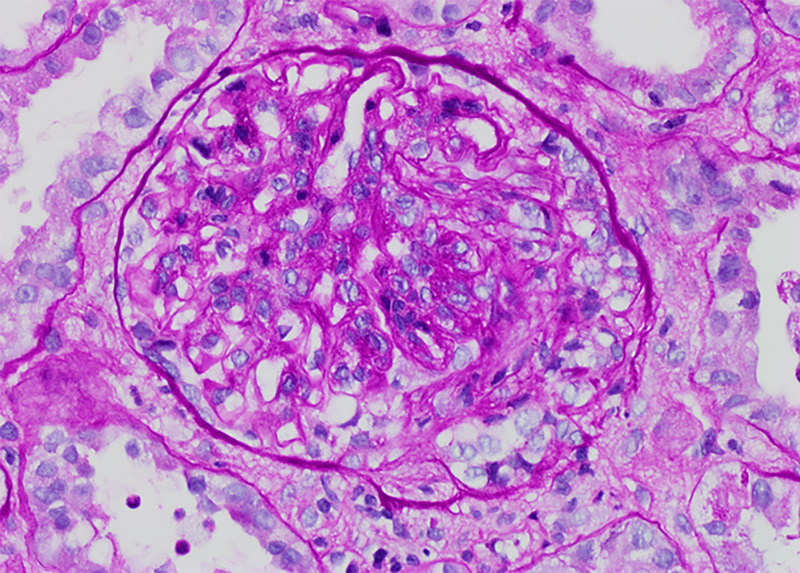
Glomeruli showing cellular crescent on renal biopsy

Simultaneously, the serum creatinine was found to be elevated at 2 mg/dL, and the patient continued to have significant proteinuria with a random urine protein creatinine ratio of 9. A second renal biopsy was performed, and it showed 75% of glomeruli with cellular crescents (Figure 5).

The patient was treated with IV cyclophosphamide, and he was started on mycophenolate while the steroids were weaned off due to neurological side effects. The neurological findings improved after weaning off of steroids.

After cyclophosphamide therapy, there was no recurrence of cardiac, GI, or neurological complications from HSP. The patient was closely followed up in the nephrology clinic. Lisinopril was added to his treatment regimen as the serum creatinine improved. Nine months after presentation, the serum creatinine was found to be 1 mg/dL; the proteinuria had improved with urine protein and creatinine ratio of 0.5, and serum albumin was normal at 3.9 g/dL. The patient was maintained on lisinopril and mycophenolate.

## Discussion

The European League Against Rheumatism (EULAR) and Pediatric Rheumatology International trials Organization (PRINTO) have published consensus guidelines for the diagnosis of HSP; according to them, a diagnosis of the condition requires the presence of palpable purpura or petechiae with lower limb prominence and not related to thrombocytopenia as a mandatory criterion along with any one of the following minor criteria - a) abdominal pain or GI bleed, b) arthritis or arthralgia, c) renal involvement and, d) histopathology: biopsy showing typical leukocytoclastic vasculitis with predominant IgA deposits or proliferative glomerulonephritis with predominant IgA deposits [5]. The biopsy is recommended for all doubtful cases of purpura and with the atypical distribution [5]. When defined as the recurrence of symptoms such as characteristic skin rash, joint pains, or GI symptoms after an asymptomatic period of two to four weeks, HSP recurrences are common with reported rates between 33% to 66% as per previous studies [6]. The risk of HSP disease recurrence, renal involvement, and severe kidney disease is high when the disease onset occurs in late childhood, adolescence, or at an age of more than 10 years, as per previous studies [7-8].

As suggested by abnormal urinalysis or elevated BUN and serum creatinine, renal involvement is often not present at disease onset, with most renal involvement cases diagnosed within six months of the initial presentation of HSP [9]. Standardized pathways that include more frequent monitoring of urinalysis and BP for the first three to four months after the initial diagnosis and less regularly until one year to detect renal involvement have been described [10]. While steroids do not prevent the occurrence of HSP nephritis or renal involvement, this condition must be diagnosed and treated early according to the severity of renal involvement as delayed treatment can cause short and long-term complications, including rapidly progressive glomerulonephritis and chronic kidney disease (CKD) [2]. The presence of IgA nephropathy is classified in the high-risk category with the presence of crescentic glomerulonephritis, with at least 50% of crescents found on renal biopsy or nephrotic syndrome and should be treated with steroids [11]. Similarly, with HSP, the Kidney Disease: Improving Global Outcomes (KDIGO) 2012 guidelines suggest that IV methylprednisolone or cyclophosphamide therapy should only be reserved for patients with >50% crescentic glomeruli and rapidly progressive glomerulonephritis with or without nephrotic syndrome; and if the child has proteinuria of >0.5 g/1.73m^2^, then they should be treated with angiotensin-converting enzyme (ACE) inhibitors for three to six months before the initiation of steroid treatment [2]. However, these guidelines are often inadequate in children with HSP, and delaying the use of steroids can lead to long-term complications such as CKD.

The use of steroids has been recommended even when rapidly proliferative glomerulonephritis is not present [2]. Our patient had significant renal involvement at presentation based on renal dysfunction and urinary findings. Even though the initial renal biopsy showed the absence of crescents, we decided to treat the patient with IV methylprednisolone because of renal dysfunction and high proteinuria seen at presentation. This report highlights the need to consider early IV methylprednisolone based on the age at which HSP is manifested (with adolescents at high risk) and by assessing the severity of glomerulonephritis based on renal dysfunction and proteinuria at presentation, and not just biopsy findings. The development of crescents may lag or may develop following recurrences. There was decent renal recovery and improved proteinuria in our patient once IV cyclophosphamide was started after the second renal biopsy showing crescents. Still, we did not employ this treatment at presentation because of the absence of crescents on the initial renal biopsy.

Elevated troponin levels or the presence of arrhythmias typically suggest vasculitic cardiac involvement with HSP [4]. Arrhythmias are a rare finding with HSP; they are reported mostly in adult patients and constitute findings of atrioventricular block, bundle branch block, ectopic atrial rhythm, and bradycardia [4,12]. The reported cardiac diseases in adults with HSP include myocardial infarction, congestive heart failure, left ventricular dilation, and myocardial necrosis [4]. One case report has described the use of rituximab to treat arrhythmias, while therapy with cyclophosphamide and methylprednisolone has been described elsewhere [4,13]. Our patient had atrial fibrillation and non-sustained ventricular tachycardia, which had not been reported previously to our knowledge. In this report, we suggest the use of methylprednisolone pulses, amiodarone, and metoprolol to treat arrhythmias.

The common GI manifestations in HSP, such as abdominal pain, GI bleed, and occult blood loss in stool, vomiting, and diarrhea, occur due to submucosal hemorrhage and edema in the bowel wall [1-3,14]. The small intestine and duodenum are commonly involved in HSP, and the endoscopy may sometimes show the presence of redness, petechiae or purpura, ulcerations, erosion, or submucosal hemorrhage [14]. Simultaneously, the biopsy has a low sensitivity to diagnosing vasculitis or detecting IgA deposition as most GI biopsies do not reach deeper blood vessels beyond the bowel wall mucosa [14]. On the contrary, the GI biopsy may distinguish other causes such as inflammatory bowel disease and eosinophilic gastroenteritis. Our patient presented with a massive lower GI bleed at the time of HSP recurrence. The colonoscopy showed a significantly lower GI bleed without any evident source. GI bleeding near Meckel’s diverticulum is difficult to diagnose on upper and lower endoscopy and colonoscopy alone. Capsule endoscopy or pill-cam endoscopy may be a better modality to diagnose such a bleed [15]. Our patient did not require laparotomy, and his GI bleeds improved with symptomatic management. GI bleed near Meckel’s diverticulum has not been previously reported with HSP to our knowledge.

The neurological involvement with HSP is infrequent (less than 1%) [16]. CNS vasculitis's common symptoms from HSP include headaches, seizures, focal neurological deficits, and visual or speech disturbances [16]. CNS vasculitis can present as edema, ischemia, infarction, or hemorrhage on brain MRI or cerebral vessel wall abnormalities on MR angiography (MRA) [16-17]. High signal intensity in sulci has been reported previously in CNS vasculitis [17]. The MRI findings suggestive of posterior reversible encephalopathy syndrome (PRES) can be present with HSP because of vasogenic cerebral edema associated with CNS vasculitis or hypertension [16]. Tremors and proximal muscle weakness may represent signs of steroid-induced myopathy and steroid toxicity. Still, the presence of upper motor neuron signs, including bilateral knee and ankle clonus, hyperreflexia, and upper extremity clonus with positive Hoffman sign prompted the evaluation for autoimmune encephalitis or vasculitis. Upper motor neuron signs have been reported previously with ANCA vasculitis but not with HSP to our knowledge [16,18]. Even though the MRA was normal and did not detect vessel wall abnormalities, the CNS vasculitis diagnosis related to HSP was made based on the unique neurological symptoms and sulcal prominence on MRI.

To summarize, we described a unique and rare presentation of HSP with varied recurrent episodes complicated by renal, GI, cardiac, and neurological manifestations. There are specific unique findings in our report that have not been previously reported with HSP to our knowledge:

1) GI bleeding from terminal ileum near Meckel’s diverticulum leading to symptomatic anemia

2) Type of arrhythmia: atrial fibrillation and non-sustained ventricular tachycardia on telemetry

3) Upper motor neuron signs

One limitation of our case report is that we could not establish the recurrent patterns of HSP, given the variable time limits and definition of HSP recurrences. The cardiac and neurological findings were near-approximated and presented within two weeks. The use of steroids did not appear to prevent recurrences of HSP in our case.

## Conclusions

The presentation of HSP may differ between children and adolescents, with adolescents at high risk of severe presentation and recurrences. This report offers insights into the manifestation and treatment of unique GI, cardiac, and neurological complications from HSP. The presence of crescents on renal biopsy may lag even with severe presentations. Treatment with steroids should not be delayed in cases of severe presentation when crescents are not present on renal biopsy.
